# KOREF_S1: phased, parental trio-binned Korean reference genome using long reads and Hi-C sequencing methods

**DOI:** 10.1093/gigascience/giac022

**Published:** 2022-03-24

**Authors:** Hui-su Kim, Sungwon Jeon, Yeonkyung Kim, Changjae Kim, Jihun Bhak, Jong Bhak

**Affiliations:** Korean Genomics Center (KOGIC), Ulsan National Institute of Science and Technology (UNIST), Ulsan 44919, Republic of Korea; Korean Genomics Center (KOGIC), Ulsan National Institute of Science and Technology (UNIST), Ulsan 44919, Republic of Korea; Department of Biomedical Engineering, College of Information and Biotechnology, Ulsan National Institute of Science and Technology (UNIST), Ulsan 44919, Republic of Korea; Clinomics Inc., Ulsan 44919, Republic of Korea; Korean Genomics Center (KOGIC), Ulsan National Institute of Science and Technology (UNIST), Ulsan 44919, Republic of Korea; Clinomics Inc., Ulsan 44919, Republic of Korea; Korean Genomics Center (KOGIC), Ulsan National Institute of Science and Technology (UNIST), Ulsan 44919, Republic of Korea; Clinomics Inc., Ulsan 44919, Republic of Korea; Korean Genomics Center (KOGIC), Ulsan National Institute of Science and Technology (UNIST), Ulsan 44919, Republic of Korea; Department of Biomedical Engineering, College of Information and Biotechnology, Ulsan National Institute of Science and Technology (UNIST), Ulsan 44919, Republic of Korea; Korean Genomics Center (KOGIC), Ulsan National Institute of Science and Technology (UNIST), Ulsan 44919, Republic of Korea; Department of Biomedical Engineering, College of Information and Biotechnology, Ulsan National Institute of Science and Technology (UNIST), Ulsan 44919, Republic of Korea; Clinomics Inc., Ulsan 44919, Republic of Korea; Personal Genomics Institute, Genome Research Foundation, Cheongju 28160, Republic of Korea

**Keywords:** Korean reference, KOREF_S1, ONT PromethION, PacBio HiFi, Hi-C, hybrid assembly

## Abstract

**Background:**

KOREF is the Korean reference genome, which was constructed with various sequencing technologies including long reads, short reads, and optical mapping methods. It is also the first East Asian multiomic reference genome accompanied by extensive clinical information, time-series and multiomic data, and parental sequencing data. However, it was still not a chromosome-scale reference. Here, we updated the previous KOREF assembly to a new chromosome-level haploid assembly of KOREF, KOREF_S1v2.1. Oxford Nanopore Technologies (ONT) PromethION, Pacific Biosciences HiFi-CCS, and Hi-C technology were used to build the most accurate East Asian reference assembled so far.

**Results:**

We produced 705 Gb ONT reads and 114 Gb Pacific Biosciences HiFi reads, and corrected ONT reads by Pacific Biosciences reads. The corrected ultra-long reads reached higher accuracy of 1.4% base errors than the previous KOREF_S1v1.0, which was mainly built with short reads. KOREF has parental genome information, and we successfully phased it using a trio-binning method, acquiring a near-complete haploid-assembly. The final assembly resulted in total length of 2.9 Gb with an N50 of 150 Mb, and the longest scaffold covered 97.3% of GRCh38’s chromosome 2. In addition, the final assembly showed high base accuracy, with <0.01% base errors.

**Conclusions:**

KOREF_S1v2.1 is the first chromosome-scale haploid assembly of the Korean reference genome with high contiguity and accuracy. Our study provides useful resources of the Korean reference genome and demonstrates a new strategy of hybrid assembly that combines ONT's PromethION and PacBio's HiFi-CCS.

## Introduction

Since the human genome reference was released in 2003, it has been updated and recently was patched in 2019 (GRCh38.p13) by the Genome Reference Consortium (GRC) [1]. Despite high completeness of GRCh38 assembly, it derives from 13 anonymous volunteers, mostly based on Caucasian and African ancestry [2]. It is the most precise and extensive among all human references constructed so far. Recently, owing to recent cost-effective sequencing methods, especially long-read methods, it has become possible to construct human personal references fast and efficiently [3]. The first Korean reference, KOREF, has been constructed in 2 types [4]. The first is KOREF_S1, which is a personal reference from an individual that is accompanied by parental *de novo* assemblies. The second one is KOREF_C, which is a consensus population reference that includes variome information of Koreans. KOREF was initiated by the Korean Ministry of Science and Technology in 2006 to generate a national genome and variome references, and currently it is jointly developed by the Genome Research Foundation, National Standard Reference Research Center, and the Korean Genomics Center at UNIST (Ulsan National Institute of Science and Technology). The first version of KOREF_S1, KOREF_S1v1.0, had a clear limitation of short reads and long-distance mapping-based approaches that resulted in a relatively low-quality assembly compared to the current GRCh38. We used Oxford Nanopore Technologies (ONT) PromethION and Pacific Biosciences (PacBio) HiFi sequencers to upgrade KOREF_S1 by using a publicly available KOREF cell line.

## Materials and Methods

### Sample preparation and genome sequencing

Sample preparation steps were followed as in the previous study [4, 5]. Human KOREF cell lines [6] were cultured at 37°C in 5% carbon dioxide in RPMI-1640 medium with 10% heat-inactivated fetal bovine serum. DNA was extracted from cells using the DNeasy Blood & Tissue kit (Qiagen, Germantown, MD, USA) following the manufacturer's instructions. Sequencing libraries for the ONT PromethION were prepared using the 1D ligation sequencing kit (SQK-LSK109, ONT, Oxford, UK) following the manufacturer's instructions. The products were quantified using the Bioanalyzer 2100 (Agilent, Santa Clara, CA, USA), and raw signals were generated by the PromethION R9.4.5 platform (PromethION, RRID:SCR_017987). Base-calling the raw signals was performed using Guppy v4.0.11 with the Flip-flop hac model.

Genomic DNA from KOREF blood samples was extracted using QIAGEN Blood & Cell Culture DNA Kit (Cat. No. 13,323). A total of 5 μg of each sample was used as input for library preparation. The SMRTbell library was constructed using the SMRTbell® Express Template Preparation Kit (101–357-000). Using the BluePippin Size selection system we removed the small fragments for a large-insert library. After sequencing primer v4 was annealed to the SMRTbell template, DNA polymerase was bound to the complex (Sequel Binding kit 2.0). We purified the complex using AMPure Purification to remove excess primer and polymerase prior to sequencing. The SMRTbell library was sequenced using single-molecule real-time (SMRT) cells (PacBio) using Sequel Sequencing Kit v2.1 and 10 hr movies were captured for each SMRT Cell 1M v2 using the Sequel II (Sequel II, RRID:SCR_017990) (PacBio) sequencing platform.

Hi-C libraries were generated using the Arima-Hic kit (A160105v01, San Diego, CA, USA). KOREF cell lines and blood samples were prepared for the construction of Hi-C libraries. Briefly, chromatin from cross-linked cells was solubilized and then digested using restriction enzymes MboI or Arima's multiple enzymes (GATC and GANTC). The digested ends were labelled using a biotinylated nucleotide, and ends were ligated to create ligation products. Ligation products were purified, fragmented, and selected by size using AMPure XP Beads. Illumina-compatible sequencing libraries were constructed on end repair, dA-tailing, and adaptor ligation using a modified workflow of the Hyper Prep kit (KAPA Biosystems, Inc.). The bead-bound libraries were amplified and purified using AMPure XP beads and sequenced using Illumina NovaSeq platform with a read length of 150 bp by Novogene (Beijing, China). Short paired-end raw reads using Illumina HiSeq 2000 platform were acquired from a previous study, accession No. SRR2204706.

For generating parental sequencing reads, we prepared samples from both of KOREF_S1’s parents. DNA was extracted from a sample of the donor's blood using DNAeasy Blood & Tissue Kit from QIAGEN according to the manufacturer's instructions. The quality and concentration of the extracted DNA were evaluated using NanoDrop™ One/OneC UV-Vis Spectrophotometer (Thermo Scientific). Library construction and whole-genome sequencing were performed by Illumina HiSeq platform (Illumina, USA) with 100-bp paired-end sequencing.

### Preprocessing of sequenced reads

The sequenced long- and short-read data underwent preprocessing steps such as adapter trimming, quality trimming, and error correction. For the long reads, adapter trimming was performed using Porechop v0.2.4 (Porechop, RRID:SCR_016967) [7] and removing reads with quality score <7 was performed using Guppy. For the short reads, adapter- and quality trimming were performed using Trimmomatic v0.39 (Trimmomatic, RRID:SCR_011848) [8], and error correction was performed using the tadpole.sh program of BBtools suite v38.26 (Bestus Bioinformaticus Tools, RRID:SCR_016968) [9].

### Trio-binning and read correction

**Figure 1:**
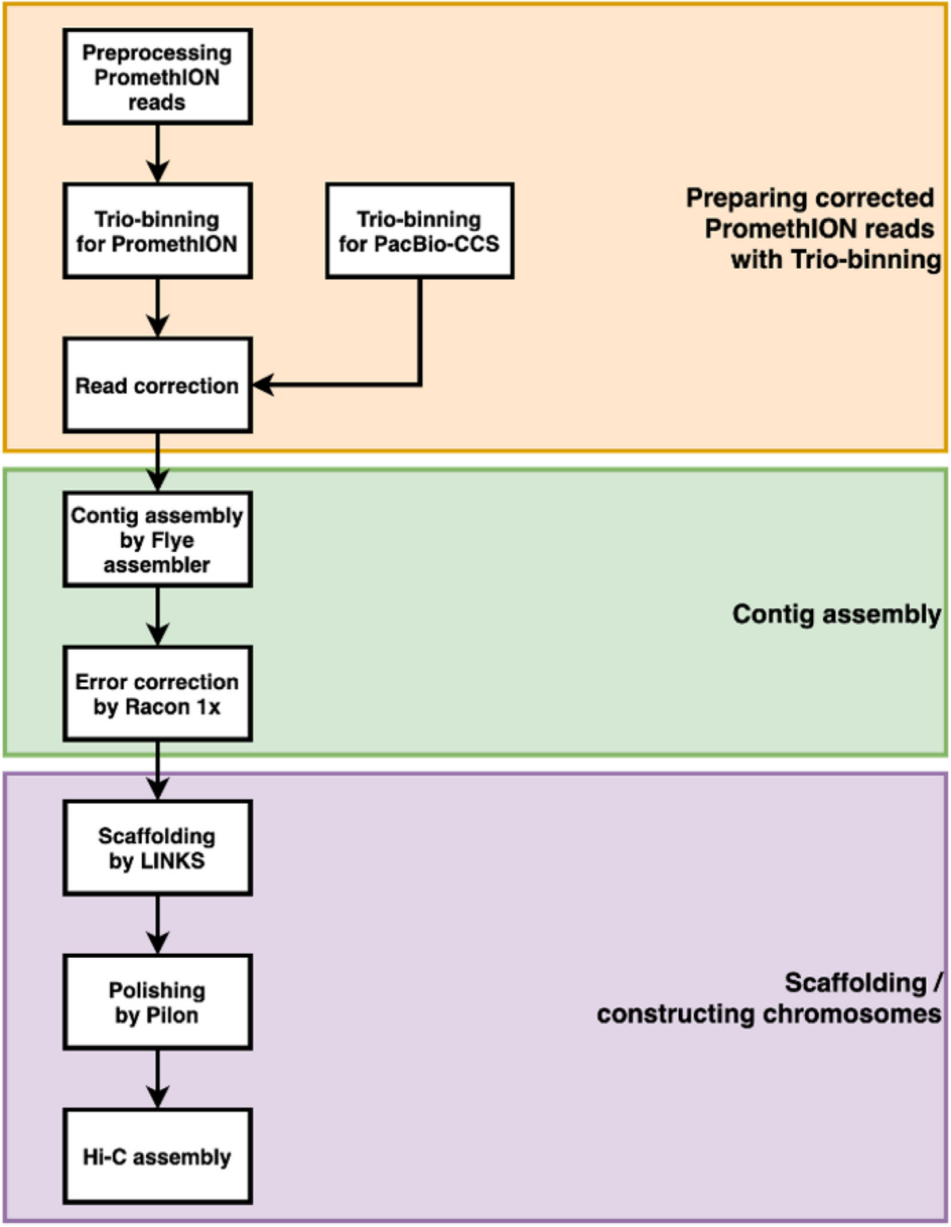
Flow chart of the KOREF reference genome assembly.

To obtain more accurate and longer haplotype-resolved reads from ONT PromethION sequencing, we applied a trio-binning with KOREF's parental sequencing data and error correction with PacBio HiFi sequencing data. The whole procedure is described in Fig. 1. To obtain haplotype-resolved reads from ONT PromethION and PacBio HiFi sequencing, we performed a trio-binning using TrioCanu v2.1 (Canu, RRID:SCR_015880) [10] with the parental short reads. In this step, reads from 11 PromethION flow-cells and 6 PacBio HiFi cells were used. We merged unclassified reads to the classified paternal and maternal reads. To correct base errors on the PromethION reads, we corrected the errors with the haplotype-resolved reads from PacBio HiFi sequencing using Racon v1.4.3 (Racon, RRID:SCR_017642) [11]. We acquired KOREF's parental sequencing data from the KOREF home page [6].

### *De novo* assembly of KOREF_S1 genome

Contig assembly was processed using wtdbg2 v2.5 [12] (WTDBG, RRID:SCR_017225) and Flye assembler v2.8.1 (Flye, RRID:SCR_017016) [13]. For the wtdbg2 assembly, parameters were set as “-x corrected -g 3g -L 5000 -X 70.0.” Error correction of the assembled contigs was conducted using Racon with a single iteration. The Flye assembly was performed with parameters of “–pacbio-hifi –hifi-error 0.008 –genome-size 3g.” For error correction, we carried out the same procedure as for the wtdbg2 assembly.

To construct scaffolds with a chromosome scale, we conducted scaffolding using PromethION reads and Hi-C data. To scaffold contigs using PromethION reads, LINKS v1.8.7 [14] was used with a single flow-cell of PromethION reads. To construct chromosome-scale scaffolds using Hi-C data, 3D-DNA pipeline v180922 [15] with the Juicer v1.6.2 program (Juicer, RRID:SCR_017226) [16] was applied to the scaffolds by LINKS. Hi-C raw reads were mapped against the extended contigs using Juicer, and the 3D-DNA pipeline was initiated to correct misjoined contigs and construct scaffolds. To correct misassemblies on the scaffolds, manual curation was performed using the JBAT (JuiceBox Assembly Tool) v1.11.08 program (Juicebox, RRID:SCR_021172) [17]. To polish base errors and small indels, we used Pilon v1.23 (Pilon, RRID:SCR_014731) [18] with KOREF's short-read data and parameters of “–fix snps and indels”.

### Constructing high-confidence regions, and the assessment of base errors on long reads and genome assemblies

To assess base errors, we constructed high-confidence regions of KOREF_S1 v1 against chromosome sequences of GRCh38.p13. The procedure was as in the study by Li et al. [19]. We aligned the KOREF_S1v1.0 assembly to GRCh38 using the Minimap2 program v2.17-r941 (Minimap2, RRID:SCR_018550) [20]. Alignments with mapping quality >5 and aligned segments shorter than 50 kb were discarded. The filtered alignments were converted to the BED format and sorted.

To assess base errors of long reads and genome assemblies, we compared them to the KOREF_S1v1.0 assembly using the assembly_assess program from Pomoxis v0.3.4 [21]. In addition, the Merqury v1.0 [22] program was used to assess assemblies using *k*-mers.

### Genome annotation

To identify protein-coding genes on the KOREF_S1v2.1 genome, we performed a liftover with a gene annotation from GENCODE 38. The liftover was processed using Liftoff v1.6.1 [23]. The result of genome annotation was stored in the KOREF genome browser, built by JBrowse v1.16.9 (JBrowse, RRID:SCR_001004) [24]. To assess protein-coding genes, BUSCO analysis (BUSCO, RRID:SCR_015008) [25] was performed using BUSCO v5.2.2 and mammalian orthoDB v10.

## Results

### KOREF_S1v2.1 assembly

We obtained 235× coverage (705 Gb) of long reads from 12 ONT PromethION flow-cells and 38× coverage (114 Gb) of long reads from 6 PacBio HiFi cells (Supplementary Table S1). We also acquired 274 Gb corrected paternal haplotype-resolved reads and 265 Gb corrected maternal haplotype-resolved reads after trio-binning and read correction. The N50 of PromethION sequencing ranged from 6,793 to 18,109 bp, and the N50 of PacBio HiFi ranged from 11,846 to 15,901 bp. Lengths of the longest read ranged from 160,294 to 1,753,381 bp for PromethION and 28,947 to 36,401 bp for PacBio HiFi. The corrected reads were identified with ∼1.4% base errors (Supplementary Table S2). Contigs from both haplotypes were assembled using wtdbg2 and Flye. The Flye assembly showed better results of higher N50 values (19.47 Mb for the paternal and 25.86 Mb for the maternal assembly) and longer length of the longest contig (87.37 Mb for the paternal and 109.79 Mb for the maternal assembly) (Table 1).

**Table 1:** Statistics of the KOREF_S1v2.1 assembly

	Contig				Scaffold	
Statistic	Wtdbg2_paternal	Flye_paternal	Wtdbg2_maternal	Flye_maternal	Paternal	Maternal
Sequence No.	3,059	2,973	2,426	2,475	2,230	2,616
Total length (bp)	2,652,350,533	2,820,210,305	2,691,371,348	2,885,670,065	2,821,407,033	2,886,600,011
N50 (bp)	15,085,508	19,472,363	15,312,743	25,861,606	141,044,433	150,051,441
Longest (bp)	70,969,653	87,371,841	70,444,093	109,786,075	235,665,501	234,237,609
Gaps (%)	0	0	0	0	0.048	0.037
GC content (%)	40.90	40.92	40.84	40.86	40.92	40.88

We extended the contigs to chromosome-scale scaffolds using 76.5 Gb of PromethION reads (Flow-cell No. 2) and 884 Gb of Hi-C data (294× sequencing depth). Scaffolds from the mitochondrial genome were excluded by using KOREF's mitochondrial DNA sequence from the previous study [4]. As a result, we acquired the paternal assembly of 2.82 Gb length with 2,230 scaffolds and an N50 of 141.04 Mb (Table 1). The maternal assembly resulted in 2,616 scaffolds with an N50 of 150.05 Mb, and its total length was 2.89 Gb. For generating the final assembly of KOREF_S1v2.1, we substituted sequences of autosomal chromosomes and a Y chromosome from the paternal assembly, and an X chromosome from the maternal assembly. As a result, KOREF_S1v2.1 was acquired with a total length of 2.9 Gb with an N50 of 150.05 Mb.

### Genome annotation

We annotated genes in KOREF_S1v2.1 by integrating a liftover of gene annotations from GENCODE release 38 [26] and homology information of RNA-sequencing data. The genes included 19,668 protein-coding genes with 85,889 transcripts, 46,973 long non-coding RNAs, and 17,535 pseudogenes (Table 3). From assessment of protein-coding genes by BUSCO, 99.3% of complete orthologous genes were found and 0.6% were missing (Table 4). A total of 1,391 genes from the Gencode38 annotation were not transferred to the KOREF by liftover, and a list of these genes can be found in Supplementary Table S4.

### Assessment of KOREF and comparison with other human genome assemblies

Using the Merqury program for quality assessment, we estimated quality value (QV) scores of Q43.88 for the paternal assembly and Q44.49 for the maternal assembly. The final assembly showed QV score of Q43.88, indicating >99.99% accuracy (Table 5), and it is higher than those of KOREF_S1v1.0 (Q33.58) and KOREF_S1v2.0 (Q39.52), which were assembled with PromethION data. We compared KOREF_S1v2.1 and other human reference genome assemblies (AK1_v2, JG2.0.0 Beta, HuRef, CHM13_v1.1, GRCh38.p13, Ash1v2.0, and PR1 v3.0) [27–32]. The results showed that KOREF_S1v2.1 has a longer scaffold N50 than AK1, HuRef, Ash1, and PR1, and scaffold N50 was comparable to JG2.0.0 Beta and CHM13_v1.1 (Table 2). Among these 8 genome assemblies, KOREF_S1v2.1 and CHM13 were the only haplotype-resolved assemblies at a chromosome scale, although KOREF_S1v2.1 has lower QV, shorter contigs, and is missing 8–10% of the human genome sequence included in CHM13_v1.1. KOREF_S1v2.1 also has longer scaffolds than recent trio-hifiasm-based assemblies but has shorter contig N50, lower QV, and substantially lower completeness. AK1 was haplotype-resolved using a read-based phasing method but could not reach chromosome scale without the guidance of a reference genome.

**Table 2:** Comparison between KOREF and other human genomes

	KOREF_S1v2.1	AK1_v2	JG2.0.0 Beta	HuRef	CHM13 v1.1	GRCh38.p13	Ash1v2.0	PR1 v3.0
Scaffolds No.	2,230	2,832	1,173	4,530	24	472	334	89
Total length (bp)	2,901,828,151	2,904,207,228	3,059,652,438	2,844,000,504	3,054,832,041	3,272,089,205	3,188,555,634	3,116,169,811
Scaffold N50 (bp)	150,051,441	44,846,623	152,668,378	143,733,266	154,259,566	67,794,783	146,254,838	149,697,505
Phasing approach	De novo	De novo	De novo	Reference-guided	De novo	De novo	Reference-guided	De novo^a^
Assembly level	Chromosome	Scaffold	Chromosome	Chromosome	Chromosome	Chromosome	Chromosome	Chromosome
Haplotype-resolved	Trio-binning	Read-based	No	No	Haploid cell line	No	No	No

a PR1 v3.0 assembly used CHM13 assembly as a reference genome to remove gaps.

**Table 3:** Statistics of the KOREF reference genome annotation

Statistic	**KOREF_S1v2.1**
Genes No.	19,668
Transcripts No.	85,889
Total length of transcripts (bp)	110,601,598
N50 (bp)	1,983
Length of longest transcripts (bp)	107,976
GC content (%)	51.60
Long non-coding RNAs No.	46,973
Pseudogenes No.	17,535

**Table 4:** Statistics of KOREF_S1v2.1 protein-coding genes using BUSCO

BUSCO assessment (%)	KOREF_S1v2.1 protein-coding genes
Complete	99.3
Complete and single-copy	40.9
Complete and duplicated	58.4
Fragmented	0.1
Missing	0.6

To identify missing regions on KOREF_S1v2.1, we made an alignment plot of KOREF against CHM13 v1.1 using Mummer v4.0.0beta2 (MUMmer, RRID:SCR_018171) [33] and Dot [34]. We found long missing sequences on centromeric regions (Supplementary Fig. S1). On chromosome 1, ∼29 Mb was missing and they were located in the centromeric region. On chromosome X, missing sequences in the centromeric region were of length ∼4 Mb (Supplementary Fig. S2).

From a pilot study of KOREF_S1’s PacBio HiFi sequencing by Hifiasm v0.15.5-r352 (Hifiasm, RRID:SCR_021069) [35], the contig assembly (KOREF_S1v2.0_PBCCS hifiasm_trio) resulted in the highest base accuracy and contiguity between HiFi-only, PromethION, and HiFi-PromethION hybrid assembly (Supplementary Table S3). The haploid completeness was found to be 99.6873% (maternal) and 99.1902% (paternal), which was 8–9% higher than that of the KOREF_S1v2.1 assembly.

To compare the assembly quality of HiFi, PromethION, and HiFi-PromethION hybrid, we compared contig assemblies from HG00744, HG002, and KOREF. The HiFi assemblies showed the highest QV and NG50 (Table 5). The HG002 assembly showed the highest QV of 51.6, and the PromethION assembly of KOREF showed the lowest QV of 33.8. The HiFi-PromethION hybrid assembly of KOREF scored higher in QV (42.2) against the PromethION assembly, but it was lower than the HiFi assembly of KOREF (QV 45.1).

**Table 5:** Comparison of contigs from HG00733, HG002, and KOREF assembly

Dataset	Sequencing platform	Assembly	Size (Gb)	QV	NG50 (Mb)
HG00733	PB HiFi	Hifiasm (trio)	6.071	49.9	34.9
HG002	PB HiFi	Hifiasm (trio)	5.967	51.6	43.0
KOREF	PB HiFi	Hifiasm (trio)	5.927	45.1	55.4
KOREF	PromethION R9.4.1	wtdbg2 (trio)	5.527	33.8	9.3
KOREF	PB HiFi—PromethION hybrid	Flye (trio)	5.706	42.2	16.5

## Discussion

In the previous version of KOREF_S1, we generated a chromosome-level genome assembly with the guidance of GRCh38. A new version of the KOREF assembly, KOREF_S1v2.1, was assembled with high accuracy (<0.01% of base error) and contiguity from multiple sequencing technologies including ONT, PacBio, Illumina, and Hi-C. Furthermore, the new KOREF assembly was phased with parental sequencing data. To generate ultra-long and highly accurate reads, we corrected the ONT reads using PacBio HiFi reads. Most genomic regions were covered by the corrected reads, but some highly competitive regions including the telomere and centromere were not covered. They had remaining gaps with unknown length. Especially on the Y chromosome, we found more gaps and less contiguity than other chromosomes. The genomic sequences of the X and Y chromosomes have highly similar regions and they probably make it difficult to phase genomic sequences on sex chromosomes.

Recently, new *de novo* assembly pipelines, such as Hifiasm [35] and HiCanu [36], have been developed for PacBio's HiFi-CCS. Hifiasm supports trio-binning from parental sequencing and Hi-C. From a pilot study by Hifiasm, a contig assembly of hifiasm_trio showed the highest base accuracy and contiguity (Supplementary Table S3). Regarding haploid completeness, it also showed the highest value, 8–9% more versus KOREF_S1v2.1. Despite these advantages, scaffolding contigs from Hifiasm has difficulties for using Hi-C data. The error-correction modules of the 3D-DNA pipeline seemed to split long repetitive sequences in a complicated fashion and made it difficult to construct scaffolds or curate misassembles (Supplementary Fig. S3). Supplementary Fig. S3A and B shows a Hi-C heat map of contigs/scaffolds without and with correcting misassemblies, respectively. In Supplementary Fig. S3A, we can find white stripe patterns from long repetitive regions, such as centromeres or telomeres, in contigs or on the border of contigs. However, in Supplementary Fig. S3B, a small number of white stripes were found in scaffolds. And we found a large amount of short contigs with long repetitive sequences that seemed to come from centromeres or telomeres. Their length totals 160 Mb. The developers of the 3D-DNA pipeline already have warned about this on their GitHub page. To avoid this problem, we needed a new strategy that made it possible to correct local misassemblies on long repetitive regions by Hi-C sequencing. However, the high-quality contigs from Hifiasm can be helpful to remove gaps and showed the possibility of resolving highly repetitive regions. Also, a recent study by the Telomere-to-Telomere (T2T) Consortium shared a complete structure of centromeric regions [30], and it will be a useful resource to complete the KOREF_S1 genome.

In conclusion, we upgraded a high-quality Korean reference genome, KOREF. Our study provides useful resources of the Korean reference genome and demonstrates a new strategy of hybrid assembly that combines use of ONT's PromethION and PacBio's HiFi-CCS.

## Data Availability

The Korean reference genome project has been deposited at DDBJ/ENA/GenBank under accession No. PRJNA735947. The version described in this article is version JAHRJT000000000. Raw DNA and RNA sequence reads for KOREF and KPGP have been submitted to the NCBI SRA database (Supplementary Table S1). The immortalized cell line of KOREF was deposited in the Korean Cell Line Bank (KCLB, No. 60211). KOREF_S1 data is available on the Korean Reference Genome Project website [37]. All supporting data and materials are available in the *GigaScience* GigaDB database [38].

## Additional Files

**Supplementary Figure S1**. Alignment of chr1 sequence, KOREF_S1v2.1 versus CHM13 v1.1.

**Supplementary Figure S2**. Alignment of chr2 sequence, KOREF_S1v2.1 versus CHM13 v1.1.

**Supplementary Figure S3**. Comparison of Hi-C heat map with and without correcting miassemblies by 3D-DNA pipeline.

**Supplementary Table S1**. The statistics of sequencing data for KOREF assembly.

**Supplementary Table S2**. Base accuracy of raw and corrected ONT long-reads.

**Supplementary Table S3**. Assessment of KOREF genome assemblies using Merqury.

**Supplementary Table S4**. The list of genes which were not partcipated in LiftOver.

giac022_GIGA-D-21-00219_Original_Submission

giac022_GIGA-D-21-00219_Revision_1

giac022_GIGA-D-21-00219_Revision_2

giac022_Response_to_Reviewer_Comments_Revision_1

giac022_Response_to_Reviewer_Comments_Revision_2

giac022_Reviewer_1_Report_Original_Submission Justin M Zook -- 8/20/2021 Reviewed

giac022_Reviewer_1_Report_Revision_1 Justin M Zook -- 12/21/2021 Reviewed

giac022_Reviewer_2_Report_Original_Submission Chaochun Wei -- 9/10/2021 Reviewed

giac022_Supplemental_File

## Abbreviations

**bp: base pairs; BUSCO: Benchmarking Universal Single-Copy Orthologs; Gb: gigabase pairs; KOREF:** KOrean REFerence; **Mb: megabase pairs; ONT:** Oxford Nanopore Technologies; PacBio: Pacific Biosciences; QV: quality value; SMRT: single-molecule real-time; SRA: Sequence Read Archive.

## Competing Interests

The authors declare that they have no competing interests.

## Funding

This work was supported by the Promotion of Innovative Businesses for Regulation-Free Special Zones funded by the Ministry of SMEs and Startups (MSS, Korea) (1425157253) (2.220037.01). This work was also supported by the Establishment of Demonstration Infrastructure for Regulation-Free Special Zones funded by the Ministry of SMEs and Startups (MSS, Korea) (1425157301) (2.220036.01). This work was also supported by the Ministry of Trade, Industry & Energy (MOTIE, Korea) under Industrial Technology Innovation Programs (“Pilot study of building of Korean Reference Standard Genome map,” No. 10046043; “Developing Korean Reference Genome,” No. 10050164; and “National Center for Standard Reference Data,” No. 10063239) and Industrial Strategic Technology Development Program (“Bioinformatics platform development for next generation bioinformation analysis,” No. 10040231).

## Authors' Contributions

J.B. supervised and coordinated the national Korean reference genome project and Personal Genome Project Korea. J.B. conceived and designed the reference genome project. H.K. performed the analyses and assembly. SJ contributed to the analysis and editing the manuscript. YK and CK performed experiments. Jihun B. contributed to bioinformatic analyses. H.K. and J.B. wrote the manuscript.
