# Correction to: KOREF_S1: Phased, parental trio-binned Korean reference genome using long reads and Hi-C sequencing methods

**DOI:** 10.1093/gigascience/giad070

**Published:** 2023-08-23

This is a correction to: Hui-su Kim and others, KOREF_S1: phased, parental trio-binned Korean reference genome using long reads and Hi-C sequencing methods, *GigaScience*, Volume 11, 2022, giac022, https://doi.org/10.1093/gigascience/giac022

In the originally published version of this manuscript, a detail was lacking in the **Funding** section. In the second sentence, this should read: “[…] funded by the Ministry of SMEs and Startups (MSS, Korea; grant numbers: P0016193)[…]” instead of: “[…] funded by the Ministry of SMEs and Startups (MSS, Korea)[…].”

This error has been corrected in the article.

