# Supplementary material for: KOREF_S1: phased, parental trio-binned Korean reference genome using long reads and Hi-C sequencing methods
Source: Gigascience. 2022 Mar 24;11:giac022. doi: 10.1093/gigascience/giac022 (PMC8952264; doi:10.1093/gigascience/giac022)
Supplement: giac022_GIGA-D-21-00219_Original_Submission [file giac022_giga-d-21-00219_original_submission.pdf]

GigaScience

KOREF_S1: the phased, parental Trio-binned Korean reference genome using long-reads and Hi-C sequencing methods

--Manuscript Draft--

Manuscript Number:	GIGA-D-21-00219
Full Title:	KOREF_S1: the phased, parental Trio-binned Korean reference genome using long-reads and Hi-C sequencing methods
Article Type:	Data Note
Funding Information:	ministry of smes and startups (P0016193) — Dr. Jong Hwa Bhak
Abstract:	Background<br><br>KOREF is the Korean reference genome which was constructed with various sequencing technologies including long reads, short reads, and optical mapping methods. It is also the first East Asian multiomic reference genome accompanied by extensive clinical information, time series and multiomic data, and his parental sequencing data. However, it was still not a chromosome-scale reference. Here, we updated the previous KOREF assembly to a new chromosome-level haploid assembly of KOREF, KOREF_S1v2.1. ONT PromethION, PacBio Hifi-CCS, and Hi-C technology were used to build the most accurate East Asian reference assembled so far.<br><br>Results<br><br>We produced 705 Gb ONT reads and 114 Gb PacBio Hifi reads, and corrected ONT reads by PacBio reads. The corrected ultra-long reads reached higher accuracy of 1.4% base-errors than the previous KOREF_S1v1.0, which was mainly built with short reads. KOREF has parental genome information, and we successfully phased it using a trio-binning method acquiring a near-complete haploid-assembly. The final assembly resulted in total length of 2.9 Gb with an N50 of 150 Mb, and the longest scaffold covered 97.3% of GRCh38's chromosome 2. And the final assembly showed high base accuracy, less than 0.01% of base-errors.<br><br>Conclusions<br><br>KOREF_S1v2.1 is the first chromosome-scale haploid assembly of the Korean reference genome with high contiguity and accuracy. Our study provides useful resources of the Korean reference genome and demonstrates a new strategy of hybrid assembly which collaborates ONT's PromethION and PacBio's HiFi-CCS.
Corresponding Author:	Jong Hwa Bhak, Ph.D.<br>UNIST<br>Ulsan, Ulsan KOREA, REPUBLIC OF
Corresponding Author Secondary Information:	
Corresponding Author's Institution:	UNIST
Corresponding Author's Secondary Institution:	
First Author:	Hui-su Kim, Ph.D.
First Author Secondary Information:	
Order of Authors:	Hui-su Kim, Ph.D.
	Sungwon Jeon
	Yeonkyung Kim
	Changjae Kim, Ph.D.

Powered by Editorial Manager® and ProduXion Manager® from Aries Systems Corporation

	Jihun Bhak
	Jong Hwa Bhak, Ph.D.
**Order of Authors Secondary Information:**	
**Additional Information:**	
**Question**	**Response**
Are you submitting this manuscript to a special series or article collection?	No
**Experimental design and statistics**<br><br>Full details of the experimental design and statistical methods used should be given in the Methods section, as detailed in our Minimum Standards Reporting Checklist. Information essential to interpreting the data presented should be made available in the figure legends.<br><br>Have you included all the information requested in your manuscript?	Yes
**Resources**<br><br>A description of all resources used, including antibodies, cell lines, animals and software tools, with enough information to allow them to be uniquely identified, should be included in the Methods section. Authors are strongly encouraged to cite Research Resource Identifiers (RRIDs) for antibodies, model organisms and tools, where possible.<br><br>Have you included the information requested as detailed in our Minimum Standards Reporting Checklist?	Yes
**Availability of data and materials**<br><br>All datasets and code on which the conclusions of the paper rely must be either included in your submission or deposited in publicly available repositories (where available and ethically appropriate), referencing such data using	Yes

Powered by Editorial Manager® and ProduXion Manager® from Aries Systems Corporation

a unique identifier in the references and in the “Availability of Data and Materials” section of your manuscript.<br><br>Have you have met the above requirement as detailed in our Minimum Standards Reporting Checklist?	

Manuscript
Click here to access/download;Manuscript;KOREF_S1v2.1_MS_giga_202107

KOREF_S1: the phased, parental Trio-binned Korean reference genome using long-reads and Hi-C sequencing methods

Hui-su Kim[1], Sungwon Jeon[1,2], Yeonkyung Kim[1], Changjae Kim[2], Jihun Bhak[1,2], and Jong Bhak[1,2,3,4]*

[1)] Korean Genomics Center (KOGIC), Ulsan National Institute of Science and Technology (UNIST), Ulsan, 44919, Republic of Korea

[2)] Department of Biomedical Engineering, College of Information and Biotechnology, Ulsan National Institute of Science and Technology (UNIST), Ulsan, 44919, Republic of Korea

[3)] Clinomics LTD, Ulsan, 44919, Republic of Korea

[4)]Personal Genomics Institute, Genome Research Foundation, Cheongju, 28160, Republic of Korea

*Correspondence author:

Name: Jong Bhak, Ph.D.

Address: #110-303, Ulsan National Institute of Science and Technology, UNIST-gil 50, Eonyang-eup, Ulju-gu, Ulsan 44919, Republic of Korea

Phone: (+82) 10-4644-6754

Email: jongbhak@genomics.org, ORCID: 0000-0002-4228-1299

Abstract

Background

KOREF is the Korean reference genome which was constructed with various sequencing technologies including long reads, short reads, and optical mapping methods. It is also the first East Asian multiomic reference genome accompanied by extensive clinical information, time series and multiomic data, and his parental sequencing data. However, it was still not a chromosome-scale reference. Here, we updated the previous KOREF assembly to a new chromosome-level haploid assembly of KOREF, KOREF_S1v2.1. ONT PromethION, PacBio Hifi-CCS, and Hi-C technology were used to build the most accurate East Asian reference assembled so far.

Results

We produced 705 Gb ONT reads and 114 Gb PacBio Hifi reads, and corrected ONT reads by PacBio reads. The corrected ultra-long reads reached higher accuracy of 1.4% base-errors than the previous KOREF_S1v1.0, which was mainly built with short reads. KOREF has parental genome information, and we successfully phased it using a trio-binning method acquiring a near-complete haploid-assembly. The final assembly resulted in total length of 2.9 Gb with an N50 of 150 Mb, and the longest scaffold covered 97.3% of GRCh38's chromosome 2. And the final assembly showed high base accuracy, less than 0.01% of base-errors.

Conclusions

KOREF_S1v2.1 is the first chromosome-scale haploid assembly of the Korean reference genome with high contiguity and accuracy. Our study provides useful resources of the Korean reference

genome and demonstrates a new strategy of hybrid assembly which collaborates ONT's PromethION and PacBio's HiFi-CCS.

Keywords: Korean reference; KOREF_S1; ONT PromethION; PacBio Hifi; Hi-C; hybrid assembly

Introduction

Since the human genome reference was released in 2003, it has been updated and recently was patched in 2019 (GRCh38.p13) by the Genome Reference Consortium (GRC) [1]. Despite high completeness of GRCh38 assembly, it derives from a single individual, mostly based on Caucasian and African ancestry [2]. It is the most precise and extensive among all human references constructed so far. Recently, due to recent cost-effective sequencing methods, especially long reads methods, one can construct human personal references fast and efficiently [3]. The first Korean reference, KOREF, has been constructed in two types [4]. The first is KOREF_S1 which is a personal reference from an individual which is accompanied by parental *de novo* assemblies. The second one is KOREF_C which is a consensus population reference that includes variome information of Koreans. KOREF was initiated by the Korean Ministry of Science and Technology in 2006 to generate a national genome and variome references and currently it is jointly developed by the Genome Research Foundation, National Standard Reference Research Center, and the Korean Genomics Center at UNIST (Ulsan National Institute of Science and Technology). The first version of KOREF_S1, KOREF_S1v1.0, had a clear limitation of short reads and long-distance mapping-based approaches that resulted in a relatively low-quality assembly compared to the current GRCh38. We used Oxford Nanopore Technologies (ONT) PromethION and PacBio HiFi sequencers to upgrade KOREF_S1 by using a publicly available KOREF cell line.

Materials and Methods

Sample preparation and genome sequencing

Sample preparation steps were followed as the previous study [4]. Human KOREF cell lines (http://koref.net) were cultured at 37°C in 5% CO_2 in RPMI-1640 medium with 10% heat-inactivated fetal bovine serum. DNA was extracted from cells using the DNeasy Blood & Tissue kit (Qiagen) to the manufacturer's instructions. Sequencing libraries for the Oxford Nanopore Technologies PromethION were prepared using the 1D ligation sequencing kit (SQK-LSK109, Oxford Nanopore Technologies, UK) following the manufacturer's instructions. The products were quantified using the Bioanalyzer 2100 (Agilent, Santa Clara, CA, USA) and raw signals were generated by the PromethION R9.4.5 platform (Oxford Nanopore Technologies, UK). Base-calling the raw signals was performed using Guppy v4.0.11 with the Flip-flop hac model.

Genomic DNA from KOREF blood samples was extracted using QIAGEN Blood & Cell Culture DNA Kit (cat no 13323). A total of 5 μg of each sample was used as input for library preparation. The SMRTbell library was constructed using the SMRTbell® Express Template Preparation Kit (101-357-000). Using the BluePippin Size selection system we removed the small fragments for a large-insert library. After sequencing primer v4 was annealed to the SMRTbell template, DNA polymerase was bound to the complex (Sequel Binding kit 2.0). We purified the complex using AMPure Purification to remove excess primer and polymerase prior to sequencing. The SMRTbell library was sequenced using SMRT cells (Pacific Biosciences) using Sequel Sequencing Kit v2.1 and 10 hr movies were captured for each SMRT Cell 1M v2 using the Sequel II (Pacific Biosciences) sequencing platform.

Hi-C libraries were generated using the Arima-Hic kit (A160105v01, San Diego, CA, USA). KOREF cell lines and blood samples were prepared for the construction of Hi-C libraries. Briefly, chromatin from cross-linked cells was solubilized and then digested using restriction enzymes MboI or Arima's multiple enzymes (GATC and GANTC). The digested ends were labeled using

a biotinylated nucleotide, and ends were ligated to create ligation products. Ligation products were purified, fragmented, and selected by size using AMPure XP Beads. Illumina-compatible sequencing libraries were constructed on end repair, dA-tailing, and adaptor ligation using a modified workflow of the Hyper Prep kit (KAPA Biosystems, Inc.). The bead-bound libraries were amplified and purified using AMPure XP beads and sequenced using Illumina NovaSeq platform with a read-length of 150 bp by Novogene (Beijing, China).

Short paired-end raw reads using Illumina HiSeq 2000 platform were acquired from a previous study, accession no. SRR2204706 (ftp://ftp.sra.ebi.ac.uk/vol1/srr/SRR220/006/SRR2204706).

For generating parental sequencing reads, we prepared samples from both of KOREF_S1's parents. DNA was extracted from the donor's blood using DNAeasy Blood & Tissue Kit from QIAGEN according to the manufacturer's instruction. The quality and concentration of the extracted DNA were evaluated using NanoDrop™ One/OneC UV-Vis Spectrophotometer (Thermo Scientific™). Library construction and whole-genome sequencing were performed by Illumina HiSeq platform (Illumina, USA) with a 100 bp paired-end sequencing.

Preprocessing of sequenced reads

The sequenced long- and short-reads data were performed preprocessing steps as adapter trimming, quality trimming, and error correction. For the long reads, adapter trimming was performed using Porechop v0.2.4 (https://github.com/rrwick/Porechop) (Porechop, RRID:SCR_016967) and removing reads with below quality-score 7 was performed using Guppy. For the short reads, adapter- and quality trimming were performed using Trimmomatic v0.39 [5] (Trimmomatic, RRID:SCR_011848), and an error correction was performed using the tadpole.sh program of

BBtools suite v38.26 (https://jgi.doe.gov/data-and-tools/bbtools) (Bestus Bioinformaticus Tools, RRID:SCR_016968).

Trio-binning and read correction

To obtain more accurate and longer haplotype-resolved reads from ONT PromethION sequencing, we applied a trio-binning with KOREF's parental sequencing data and an error-correction with PacBio Hifi sequencing data. The whole procedure is described in figure 1. To obtain haplotype-resolved reads from ONT PromethION and PacBio Hifi sequencing, we performed a trio-binning using TrioCanu v2.1 [6] (Canu, RRID:SCR_015880) with the parental short-reads. In this step, reads from eleven PromethION flow-cells and six PacBio Hifi cells were participated. We merged unclassified reads to the classified paternal-reads and classified maternal-reads each. To correct base-errors on the PromethION reads, we corrected the errors with the haplotype-resolved reads from PacBio Hifi sequencing using Racon v1.4.3 [7] (Racon, RRID:SCR_017642). We acquired KOREF's parental sequencing data from the KOREF homepage (http://koref.net/KOREF_Data_Download).

De novo assembly of KOREF_S1 genome

Contig assembly was processed using wtdbg2 v2.5 [8] (WTDBG, RRID:SCR_017225) and Flye assembler v2.8.1 [9] (Flye, RRID:SCR_017016). For a wtdbg2 assembly, parameters were set as '-x corrected -g 3g -L 5000 -X 70.0'. An error correction of the assembled contigs was conducted using Racon with a single iteration. The Flye assembly was performed with parameters of '--

pacbio-hifi --hifi-error 0.008 --genome-size 3g'. For error correction, we carried out the same procedure as the wtdbg2 assembly.

To construct scaffolds with a chromosome-scale, we conduct scaffolding using PromethION reads and Hi-C data. To scaffold contigs using PromethION reads, LINKS v1.8.7 [10] was used with a single flow-cell of PromethION reads. To construct chromosome-scale scaffolds using Hi-C data. 3D-DNA pipeline v180922 [11] with Juicer v1.6.2 program [12] (Juicer, RRID:SCR_017226) was performed with the scaffolds by LINKS. Hi-C raw reads were mapped against the extended contigs using Juicer, and the 3D-DNA pipeline was initiated to correct mis-joined contigs and construct scaffolds. To correct misassemblies on the scaffolds, a manual curation was performed using JBAT (JuiceBox Assembly Tool) v1.11.08 program (https://github.com/aidenlab/Juicebox) (Juicebox, RRID:SCR_021172). To polish base-errors and small indels, we performed Pilon v1.23 program [13] (Pilon, RRID:SCR_014731) with KOREF's short read data and parameters of '--fix snps and indels' were used.

Constructing high-confident regions, and the assessment of base-errors on long-reads and genome assemblies

For an assessment of base-errors, we constructed high-confident regions of KOREF_S1 v1 against chromosome sequences of the GRCh38.p13. The procedure was referred to Heng Li's study [14]. We aligned the KOREF_S1v1.0 assembly to GRCh38 using the Minimap2 program v2.17-r941 [15] (Minimap2, RRID:SCR_018550). Alignments with mapping quality >5 and aligned segments shorter than 50 kb were discarded. The filtered alignments were converted to the BED format and sorted.

To assess base-errors of long-reads and genome assemblies, we compared them to the KOREF_S1v1.0 assembly using the assembly_assess program from Pomoxis v0.3.4 (https://github.com/nanoporetech/pomoxis). And the Merqury v1.0 [16] program was performed to assess assemblies using k-mers.

Genome annotation

To identify protein coding genes on KOREF_S1v2.1 genome, we performed a reference-guided transcriptome assembly with liftover with a gene annotation from GENCODE 38. The liftover was processed using Liftoff v1.6.1 program [17], and the reference-guided assembly was performed using Stringtie v2.1.5 program [18] (Stringtie, RRID:SCR_016323) and TransDecoder v5.5.0 program (https://github.com/TransDecoder/TransDecoder) (TransDecoder, RRID:SCR_017647) with KOREF's RNASeq data. Mapping RNASeq data was conducted using HISAT2 v2.2.1 program [19] (HISAT2, RRID:SCR_015530) for short reads and GMAP v2020-06-01 program [20] (GMAP, RRID:SCR_008992) for PacBio ISOSeq reads. Other genes including lncRNAs and pseudo-genes were annotated by the liftover. The result of genome annotation was stored in the KOREF genome browser, built by the JBrowse v1.16.9 [21] (JBrowse, RRID:SCR_001004). All RNASeq data were collected from the KOREF homepage (http://koref.net/KOREF_Data_Download).

Results

KOREF_S1v2.1 assembly

We obtained 235× coverage (705 Gb) of long-reads from twelve ONT PromethION flow-cells and 38× coverage (114 Gb) of long reads from six PacBio HiFi cells (Table S1). We also acquired 274 Gb corrected paternal haplotype-resolved reads and 265 Gb corrected maternal haplotype-resolved reads after trio-binning and read-correction. The corrected reads were identified about 1.4% base-errors (Table S2). Contigs from both haplotypes were assembled using wtdbg and Flye. The Flye assembly showed better results of higher N50 values (19.47 Mb for a paternal and 25.86 Mb for a maternal assembly) and longer length of the longest contig (70.97 Mb for a paternal and 109.79 Mb for a maternal assembly) (Table 1).

We extended the contigs to chromosome-scale scaffolds using 76.5 Gb of PromethION reads (Flow-cell no.2) and 884 Gb of Hi-C data (294× sequencing-depth). Scaffolds from a mitochondrial genome were excluded using the KOREF's mtDNA sequence from the previous study [4]. As a result, we acquired the paternal assembly of 2.82 Gb length with 2,230 scaffolds and an N50 of 141.04 Mb (Table 1). The maternal assembly resulted in 2,616 scaffolds with an N50 of 150.05 Mb, and its total length was 2.88 Gb. For generating the final assembly of KOREF_S1v2.1, we substituted sequences of autosomal chromosomes and a Y chromosome from the paternal assembly, and a X chromosome from the maternal assembly. As a result, the KOREF_S1v2.1 was acquired a total length of 2.9 Gb with an N50 of 150.05 Mb.

Genome annotation

We annotated genes in KOREF_S1v2.1 by integrating a liftover of gene annotations from the GENCODE release 38 (https://www.gencodegenes.org/human/) and homology information of RNASeq data. The genes included 20,378 protein-coding genes with 166,570 transcripts, 46,973

lncRNAs and 17,535 pseudogenes (Table 3). 1,391 genes from the Gencode38 annotation were not transferred to the KOREF by liftover, and a list of these genes can be found in the supplementary table 4.

Assessment of KOREF and comparison with other human genome assemblies

Using the Merqury program for a quality assessment, we estimated QV scores of Q43.88 for the paternal assembly and Q44.49 for the maternal assembly. The final assembly showed QV score of Q43.88, indicating >99.99% accuracy (Table S5), and it is higher than KOREF_S1v1.0's (Q33.58) and KOREF_S1v2.0 (Q39.52) which was were assembled with the PromethION data. We compared KOREF_S1v2.1 and other human reference genome assemblies (AK1_v2, JG2.0.0 Beta, HuRef, CHM13_v1.1, and GRCh38.p13) [22-25]. The results showed that KOREF_S1v2.1 is more contiguous than AK1 and HuRef, and comparable to JG2.0.0 Beta and CHM13_v1.1 (Table 2). Among six genome assemblies, KOREF_S1v2.1 and CHM13 were a haplotype-resolved assembly with a chromosome-scale. AK1 was haplotype-resolved using a read-based phasing method but could not reach a chromosome-scale without a guidance of the reference genome.

Discussion

In previous version of KOREF_S1, we generated a chromosome-level genome assembly with a guidance of GRCh38. A new version of KOREF assembly, KOREF_S1v2.1, was assembled with high accurate (less than 0.01% of base error) and contiguity from multiple sequencing technologies including ONT, PacBio, Illumina, and Hi-C. Furthermore, the new KOREF assembly was phased

with parental sequencing data. To generate ultra-long and high accurate reads, we corrected ONT reads using PacBio HiFi reads. Most genomic regions were covered by the corrected reads, but some highly competitive regions including telomere and centromere were not covered. They were remained as gaps with unknown length. Especially on a chromosome Y, we found more gaps and less contiguity than other chromosomes. The genomic sequences of a chromosome X and Y have high similar regions and they probably make difficulties to phase genomic sequences on sex chromosomes.

Recently, new *de novo* assembly pipelines, such as the Hifiasm [26] (Hifiasm, RRID:SCR_021069) and HiCanu [27], have been developed for PacBio's HiFi-CCS. Especially, Hifiasm supports a trio-binning from parental sequencing and Hi-C, and the assembly resulted in high base-accuracy and contiguity (Table S3). Despite these advantages, scaffolding contigs from Hifiasm has difficulties for using Hi-C data. From a pilot study, an error-correction module of the 3D-DNA pipeline seemed to split long repetitive regions complicatedly, and it made difficult to construct scaffolds or curate misassembles (Fig. S1). However, the high-quality contigs from Hifiasm can be helpful to remove gaps and resolve highly repetitive regions. Also, a recent study of the T2T consortium shared a complete structure of centromeric regions [25], and it will be a useful resource to complete the KOREF_S1 genome.

In conclusion, we upgraded a high-quality Korean reference genome, KOREF. Our study provides useful resources of the Korean reference genome and demonstrates a new strategy of hybrid assembly which collaborates ONT's PromethION and PacBio's HiFi-CCS.

Data availability

The Korean reference genome project has been deposited at DDBJ/ENA/GenBank under the accession PRJNA735947. The version described in this paper is version JAHRJT000000000. Raw DNA and RNA sequence reads for KOREF and KPGP have been submitted to the NCBI Sequence Read Archive database (from SRR14759111 to SRR14759134). The immortalized cell line of KOREF was deposited in the Korean Cell Line Bank (KCLB, #60211). KOREF_S1 data is found from http://koreanreference.org

Competing financial interests

The authors declare no competing financial interests.

Funding

This work was supported by the Promotion of Innovative Businesses for Regulation-Free Special Zones funded by the Ministry of SMEs and Startups (MSS, Korea)(P0016193).

Author contributions

J.B. supervised and coordinated the national Korean reference genome project and Personal Genome Project Korea. J.B. conceived and designed the reference genome project. H.K. performed the analyses and assembly. H.K. and J.B. wrote the manuscript.

Acknowledgements

This work was supported by the Ministry of Trade, Industry & Energy (MOTIE, Korea) under Industrial Technology Innovation Programs ('Pilot study of building of Korean Reference Standard Genome map', No.10046043; 'Developing Korean Reference Genome', No.10050164; and 'National Center for Standard Reference Data', No.10063239) and Industrial Strategic Technology Development Program ('Bioinformatics platform development for next generation bioinformation analysis', No.10040231). Korea Institute of Science and Technology Information (KISTI) provided us with Korea Research Environment Open NETwork (KREONET) which is the internet connection service for efficient information and data transfer. We thank Jaesu Bhak for editing the manuscript.

References

1. Schneider VA, Graves-Lindsay T, Howe K, Bouk N, Chen HC, Kitts PA, et al. Evaluation of GRCh38 and de novo haploid genome assemblies demonstrates the enduring quality of the reference assembly. Genome Res. 2017;27 5:849-64. doi:10.1101/gr.213611.116.
2. Green RE, Krause J, Briggs AW, Maricic T, Stenzel U, Kircher M, et al. A Draft Sequence of the Neandertal Genome. Science. 2010;328 5979:710-22. doi:10.1126/science.1188021.
3. Logsdon GA, Vollger MR and Eichler EE. Long-read human genome sequencing and its applications. Nat Rev Genet. 2020;21 10:597-614. doi:10.1038/s41576-020-0236-x.
4. Cho YS, Kim H, Kim HM, Jho S, Jun J, Lee YJ, et al. An ethnically relevant consensus Korean reference genome is a step towards personal reference genomes. Nat Commun. 2016;7 doi:ARTN 13637 10.1038/ncomms13637.
5. Bolger AM, Lohse M and Usadel B. Trimmomatic: a flexible trimmer for Illumina sequence data. Bioinformatics. 2014;30 15:2114-20. doi:10.1093/bioinformatics/btu170.
6. Koren S, Rhie A, Walenz BP, Dilthey AT, Bickhart DM, Kingan SB, et al. De novo assembly of haplotype-resolved genomes with trio binning. Nat Biotechnol. 2018; doi:10.1038/nbt.4277.
7. Vaser R, Sovic I, Nagarajan N and Sikic M. Fast and accurate de novo genome assembly from long uncorrected reads. Genome Res. 2017;27 5:737-46. doi:10.1101/gr.214270.116.
8. Ruan J and Li H. Fast and accurate long-read assembly with wtdbg2. Nat Methods. 2020;17 2:155-+. doi:10.1038/s41592-019-0669-3.

9. Kolmogorov M, Yuan J, Lin Y and Pevzner PA. Assembly of long, error-prone reads using repeat graphs. Nat Biotechnol. 2019;37 5:540-+. doi:10.1038/s41587-019-0072-8.
10. Warren RL, Yang C, Vandervalk BP, Behsaz B, Lagman A, Jones SJM, et al. LINKS: Scalable, alignment-free scaffolding of draft genomes with long reads. Gigascience. 2015;4 doi:ARTN 35 10.1186/s13742-015-0076-3.
11. Dudchenko O, Batra SS, Omer AD, Nyquist SK, Hoeger M, Durand NC, et al. De novo assembly of the Aedes aegypti genome using Hi-C yields chromosome-length scaffolds. Science. 2017;356 6333:92-5. doi:10.1126/science.aal3327.
12. Durand NC, Shamim MS, Machol I, Rao SS, Huntley MH, Lander ES, et al. Juicer Provides a One-Click System for Analyzing Loop-Resolution Hi-C Experiments. Cell Syst. 2016;3 1:95-8. doi:10.1016/j.cels.2016.07.002.
13. Walker BJ, Abeel T, Shea T, Priest M, Abouelliel A, Sakthikumar S, et al. Pilon: An Integrated Tool for Comprehensive Microbial Variant Detection and Genome Assembly Improvement. Plos One. 2014;9 11 doi:ARTN e112963 10.1371/journal.pone.0112963.
14. Li H, Bloom JM, Farjoun Y, Fleharty M, Gauthier L, Neale B, et al. A synthetic-diploid benchmark for accurate variant-calling evaluation. Nat Methods. 2018;15 8:595-7. doi:10.1038/s41592-018-0054-7.
15. Li H. Minimap2: pairwise alignment for nucleotide sequences. Bioinformatics. 2018;34 18:3094-100. doi:10.1093/bioinformatics/bty191.
16. Shumate A and Salzberg SL. Liftoff: accurate mapping of gene annotations. Bioinformatics. 2020; doi:10.1093/bioinformatics/btaa1016.
17. Pertea M, Pertea GM, Antonescu CM, Chang TC, Mendell JT and Salzberg SL. StringTie enables improved reconstruction of a transcriptome from RNA-seq reads. Nat Biotechnol. 2015;33 3:290-5. doi:10.1038/nbt.3122.
18. Kim D, Paggi JM, Park C, Bennett C and Salzberg SL. Graph-based genome alignment and genotyping with HISAT2 and HISAT-genotype. Nat Biotechnol. 2019;37 8:907-15. doi:10.1038/s41587-019-0201-4.
19. Wu TD and Watanabe CK. GMAP: a genomic mapping and alignment program for mRNA and EST sequences. Bioinformatics. 2005;21 9:1859-75. doi:10.1093/bioinformatics/bti310.
20. Buels R, Yao E, Diesh CM, Hayes RD, Munoz-Torres M, Helt G, et al. JBrowse: a dynamic web platform for genome visualization and analysis. Genome Biol. 2016;17:66. doi:10.1186/s13059-016-0924-1.
21. Rhie A, Walenz BP, Koren S and Phillippy AM. Merqury: reference-free quality, completeness, and phasing assessment for genome assemblies. Genome Biol. 2020;21 1:245. doi:10.1186/s13059-020-02134-9.
22. Seo JS, Rhie A, Kim J, Lee S, Sohn MH, Kim CU, et al. De novo assembly and phasing of a Korean human genome. Nature. 2016;538 7624:243-+. doi:10.1038/nature20098.
23. Takayama J, Tadaka S, Yano K, Katsuoka F, Gocho C, Funayama T, et al. Construction and integration of three de novo Japanese human genome assemblies toward a population-specific reference. Nat Commun. 2021;12 1:226. doi:10.1038/s41467-020-20146-8.
24. Levy S, Sutton G, Ng PC, Feuk L, Halpern AL, Walenz BP, et al. The diploid genome sequence of an individual human. PLoS Biol. 2007;5 10:e254. doi:10.1371/journal.pbio.0050254.

25. Cheng H, Concepcion GT, Feng X, Zhang H and Li H. Haplotype-resolved de novo assembly using phased assembly graphs with hifiasm. Nat Methods. 2021;18 2:170-5. doi:10.1038/s41592-020-01056-5.
26. Nurk S, Walenz BP, Rhie A, Vollger MR, Logsdon GA, Grothe R, et al. HiCanu: accurate assembly of segmental duplications, satellites, and allelic variants from high-fidelity long reads. Genome Res. 2020;30 9:1291-305. doi:10.1101/gr.263566.120.
27. Nurk S, Koren S, Rhie A, Rautiainen M, Bzikadze AV, Mikheenko A, et al. The complete sequence of a human genome. bioRxiv. 2021.

Figures

Figure 1. The flowchart of KOREF genome assembly

Tables

Table 1. The statistics of KOREF_S1v2.1 assembly

	Contig				Scaffold	
	Wtdbg2_paternal	Flye_paternal	Wtdbg2_maternal	Flye_maternal	Paternal	Maternal
Sequence no.	3,059	4,463	2,426	2,475	2,230	2,616
Total length (bp)	2,652,350,533	2,820,210,305	2,691,371,348	2,885,670,065	2,821,407,033	2,886,600,011
N50 (bp)	15,085,508	19,472,363	15,312,743	25,861,606	141,044,433	150,051,441
Longest (bp)	70,969,653	87,371,841	70,444,093	109,786,075	235,665,501	234,237,609
Gaps	0.000%	0.000%	0.000%	0.000%	0.048%	0.037%
GC contents	40.90%	40.92%	40.84%	40.86%	40.92%	40.88%

Table 2. Comparison between KOREF and other human genomes

	KOREF_S1v2.1	AK1_v2	JG2.0.0 Beta	HuRef	CHM13	GRCh38.p13
Scaffold no.	2,230	2,832	1,173	4,530	24	472
Total length (bp)	2,901,828,151	2,904,207,228	3,059,652,438	2,844,000,504	3,054,832,041	3,272,089,205
Scaffold N50 (bp)	150,051,441	44,846,623	152,668,378	143,733,266	154,259,566	67,794,783
Phasing approach	*De novo*	*De novo*	*De novo*	Reference-guided	*De novo*	*De novo*
Assembly level	Chromosome	Scaffold	Chromosome	Chromosome	Chromosome	Chromosome
Haplotype-resolved	Trio-binning	Read-based	No	No	Haploid cell line	No

Table 3. The statistics of KOREF genome annotation

	**KOREF_S1v2.1 gene**
Genes no.	20,378
Transcripts no.	166,570
Total length of transcripts (bp)	216,532,041
N50 (bp)	1,851
Length of longest transcripts	107,976
GC contents	52.22%
lncRNAs no.	46,973
Psuedogenes no.	17,535

Figure 1

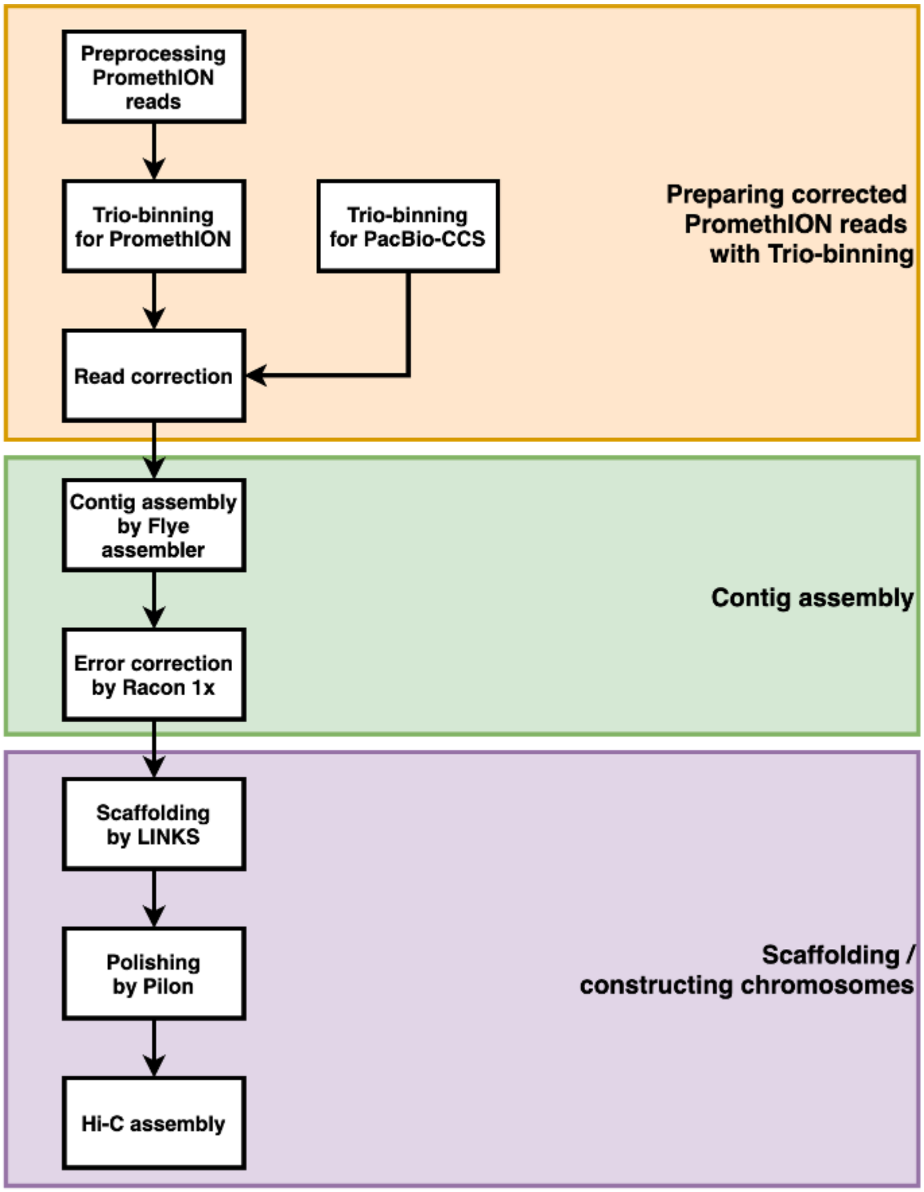
Preprocessing PromethION reads
Trio-binning for PromethION
Trio-binning for PacBio-CCS
Read correction
Preparing corrected PromethION reads with Trio-binning
Contig assembly by Flye assembler
Error correction by Racon 1x
Contig assembly
Scaffolding by LINKS
Polishing by Pilon
Hi-C assembly
Scaffolding / constructing chromosomes

Supplementary tables

Click here to access/download
Supplementary Material
KOREF_S1v2.1_supplementary_tables.20210726.xlsx
