# Supplementary material for: KOREF_S1: phased, parental trio-binned Korean reference genome using long reads and Hi-C sequencing methods
Source: Gigascience. 2022 Mar 24;11:giac022. doi: 10.1093/gigascience/giac022 (PMC8952264; doi:10.1093/gigascience/giac022)
Supplement: giac022_Response_to_Reviewer_Comments_Revision_2 [file giac022_response_to_reviewer_comments_revision_2.pdf]

Author's Response To Reviewer Comments

Close

Revision for KOREF manuscript to the GigaScience

Reviewer #1:
The authors present an improved reference assembly for an extensively characterized Korean son in a trio. Specifically, they partition ONT and HiFi reads by haplotype, correct ONT reads with HiFi reads, and assemble the corrected reads followed by scaffolding with Hi-C. This is an assembly approach I haven't seen before, and it yields impressive chromosome-scale scaffolds. However, the completeness, contig N50's, and QV's are substantially worse than recent assemblies from HiFi data alone, particularly from trio-hifiasm,

↳ We noted and emphasized the limitation of our assembly.

so I think the authors need to better emphasize the limitations of their assembly, as well as its strengths. If this is made clear, I expect this to be a useful manuscript.

1. The authors should clearly state in the results that their QV of ~44 is substantially lower than the QV of ~50 for recently published hifiasm assemblies that use HiFi data alone, albeit HiFi with longer read lengths (https://www.nature.com/articles/s41592-020-01056-5/tables/3)

↳ Thank you. It is true that our QV was substantially lower than the QV of Hifiasm assemblies. We have now additionally compared contig assemblies of KOREF, HG00733, and HG002. All results are in Table 5. HG002 assembly showed highest QV of 51.6 and PromethION assembly of KOREF showed lowest QV of 33.8. HiFi-PromethION hybrid assembly of KOREF scored higher QV (42.2) against PromethION assembly. However, it was lower than the HiFi assembly of KOREF (QV 45.1). We noted this on Line 303.

2. The authors should clearly state in the main text that their assembly's completeness in Table S3 is only 90-92%, >10x more missing sequence than their hifiasm assembly (99.2-99.7%)

↳ We agree. The hifiasm assemblies showed 8~9% higher than HiFi-PromethION hybrid assembly on haploid completeness. We stated this on the discussion section, line 325.

3. Could the authors use their dipcall analysis to better understand what is missing from the assembly (e.g., segmental duplications)?

↳ To identify missing regions, we made an alignment of our assembly against CHM13 v1.1 using Mummer and Dot. From an alignment against CHM13, we found long missing sequences on centromeric regions and they could be found on Fig. S1 (chr. 1) and S2 (chr. X).

4. I suspect the assembly may collapse many segmental duplications, causing base-level and structural errors in the assembly, which could cause many problems when using the reference, so the authors should make this clear. For example, how many of the missing genes are in segmental duplications?

↳ From an alignment against CHM13, we found long missing sequences on centromeric regions and they could be found on Fig. S1 (chr. 1) and S2 (chr. X). On chromosome one, about 29 Mb was missing and they were located on a centromeric region. On chromosome X, missing sequences of a centromeric region had a length of about 4 Mb (Fig. S2). We stated this on Line 242.

5. What version of hifiasm was used by the authors?

↳ We used v0.15.5-r352.

6. This statement is mis-leading, since the CHM13 reference is much more complete and contiguous,

even though KOREF has a comparable scaffold length: "The results showed that KOREF_S1v2.1 is more contiguous than AK1 and HuRef, and comparable to JG2.0.0 Beta and CHM13_v1.1 (Table 2). Among six genome assemblies, KOREF_S1v2.1 and CHM13 were a haplotype-resolved assembly with a chromosome-scale". It should be revised to something like "The results showed that KOREF_S1v2.1 has longer scaffold N50 than AK1 and HuRef, and scaffold N50 comparable to JG2.0.0 Beta and CHM13_v1.1 (Table 2). Among these six genome assemblies, KOREF_S1v2.1 and CHM13 were the only haplotype-resolved assemblies with a chromosome-scale, though KOREF_S1v2.1 has lower QV, shorter contigs, and is missing 8-10% of the human genome sequence included in CHM13_v1.1. KOREF_S1v2.1 also has longer scaffolds than recent trio-hifiasm-based assemblies, but has shorter contig N50, lower QV, and substantially lower completeness."

↳ We agree with your comments and revised the texts according to your suggestion.

7. Could the authors please elaborate on this conclusion "From a pilot study, an error-correction module of the 3D-DNA pipeline seemed to split long repetitive regions complicatedly, and it made difficult to construct scaffolds or curate misassembles (Fig. S1)"? No Fig S1 was included in the submission, and this merits more discussion and detailed methods if the authors want to claim this.

↳ You are right. We missed to include Fig. S1 and prepared it as Fig. S3-A and -B. We constructed scaffolds using contigs from KOREF's paternal hifiasm assembly and Hi-C sequencing data by 3D-DNA pipeline. Fig. S3-A shows a Hi-C heat map of contigs without correcting misassemblies and Fig. S3-B shows a Hi-C heat map of contigs/scaffolds with correcting misassemblies. On Fig. S3-A, we can find white stripe patterns from long repetitive regions, such as centromeres or telomeres, in contigs or on the border of contigs. However, on Fig. S3-B, a small number of white stripes were found in scaffolds. And we found a large amount of short contigs with long repetitive sequences that have appeared to come from centromeres or telomeres. Its length reaches 160 Mb. The developers of 3D-DNA pipeline already have warned this on their github page. To avoid this problem, we needed to build a new strategy that enabled to correct local misassemblies on long repetitive regions by Hi-C sequencing. We noted this on Line 286.
8. The authors should state in the main text that the N50 read lengths from ONT and HiFi, since they are relatively small compared to current best practice.

↳ Good point. We added N50 read lengths and the longest read lengths from ONT and HiFi in the results section (Line 180). An N50 of PromethION sequencing ranged from 6,793 bp to 18,109 bp and an N50 of PacBio HiFi ranged from 11,846 bp to 15,901. About lengths of the longest read, PromethION ranged from 160,294 bp to 1,753,381 bp and PacBio HiFi ranged from 28,947 bp to 36,401 bp.

9. It would be useful to compare to other recent reference genomes and assemblies, such as https://genomebiology.biomedcentral.com/articles/10.1186/s13059-020-02047-7, https://doi.org/10.1101/2021.06.10.447952, and https://www.nature.com/articles/s41592-020-01056-5

↳ Thank you for recommending additional human genome assemblies. We have now added some comparison statistics of Ash1 assembly and PR1 assembly to table 2 (Line 231). The results showed that KOREF_S1v2.1 has longer scaffold N50 than AK1, HuRef, Ash1 and PR1, and scaffold N50 was comparable to JG2.0.0 Beta and CHM13_v1.1.

Reviewer #2:
This paper reported the construction of KOREF_S1v2, a reference genome for Korean or Eastern Asian, using long read sequencing platforms in addition to NGS sequencing and HiC sequencing platforms. A reference genome construction method was introduced to combine parent genomes to increase the quality of the final assembled genome. The constructed genome was assessed for its quality by comparing it to the existing KOREF genome and the human reference genome. The goal of this paper is to provide an accurate Korean reference genome. A few issues are listed below.

Major issues
1. Line 52, "GRCh38 … derives from a single individual, mostly based on Caucasian and African ancestry", the content was incorrect, and the sentence needs a revision.

↳ You are right. GRCh38 was constructed from thirteen anonymous volunteers. We corrected the Line 52 from "a single individual" to "thirteen anonymous volunteers'. Thank you.

2. Line 200, "the genes included 20,378 protein-coding genes with 166,570 transcripts, 46,973 lncRNAs and 17,535 pseudogenes.", the number of protein coding transcripts, 166,570, was much bigger than the number for protein-coding transcripts listed in GENCODE, which is about 87K. Please double check the numbers.

↳ We agree with you. We found a mistake on the liftover and have fixed it. Now, we have 19,668 protein coding genes with 85,889 transcripts (Line 217). Thank you.

Minor issues

1. Line 234, "and it made difficult to construct" -->" and made it difficult to construct".

↳ Thank you. We fixed the text as your suggestion.

Close
