# Supplementary material for: KOREF_S1: phased, parental trio-binned Korean reference genome using long reads and Hi-C sequencing methods
Source: Gigascience. 2022 Mar 24;11:giac022. doi: 10.1093/gigascience/giac022 (PMC8952264; doi:10.1093/gigascience/giac022)
Supplement: giac022_Reviewer_1_Report_Original_Submission — Justin M Zook -- 8/20/2021 Reviewed [file giac022_reviewer_1_report_original_submission.pdf]

Reviewer Report

Title: KOREF_S1: the phased, parental Trio-binned Korean reference genome using long-reads and Hi-C sequencing methods

Version: Original Submission **Date:** 8/20/2021

Reviewer name: Justin M Zook

Reviewer Comments to Author:

The authors present an improved reference assembly for an extensively characterized Korean son in a trio. Specifically, they partition ONT and HiFi reads by haplotype, correct ONT reads with HiFi reads, and assemble the corrected reads followed by scaffolding with Hi-C. This is an assembly approach I haven't seen before, and it yields impressive chromosome-scale scaffolds. However, the completeness, contig N50's, and QV's are substantially worse than recent assemblies from HiFi data alone, particularly from trio-hifiasm, so I think the authors need to better emphasize the limitations of their assembly, as well as its strengths. If this is made clear, I expect this to be a useful manuscript.

1. The authors should clearly state in the results that their QV of ~44 is substantially lower than the QV of ~50 for recently published hifiasm assemblies that use HiFi data alone, albeit HiFi with longer read lengths (https://www.nature.com/articles/s41592-020-01056-5/tables/3)
2. The authors should clearly state in the main text that their assembly's completeness in Table S3 is only 90-92%, >10x more missing sequence than their hifiasm assembly (99.2-99.7%)
3. Could the authors use their dipcall analysis to better understand what is missing from the assembly (e.g., segmental duplications)?
4. I suspect the assembly may collapse many segmental duplications, causing base-level and structural errors in the assembly, which could cause many problems when using the reference, so the authors should make this clear. For example, how many of the missing genes are in segmental duplications?
5. What version of hifiasm was used by the authors?
6. This statement is mis-leading, since the CHM13 reference is much more complete and contiguous, even though KOREF has a comparable scaffold length: "The results showed that KOREF_S1v2.1 is more contiguous than AK1 and HuRef, and comparable to JG2.0.0 Beta and CHM13_v1.1 (Table 2). Among six genome assemblies, KOREF_S1v2.1 and CHM13 were a haplotype-resolved assembly with a chromosome-scale". It should be revised to something like "The results showed that KOREF_S1v2.1 has longer scaffold N50 than AK1 and HuRef, and scaffold N50 comparable to JG2.0.0 Beta and CHM13_v1.1 (Table 2). Among these six genome assemblies, KOREF_S1v2.1 and CHM13 were the only haplotype-resolved assemblies with a chromosome-scale, though KOREF_S1v2.1 has lower QV, shorter contigs, and is missing 8-10% of the human genome sequence included in CHM13_v1.1. KOREF_S1v2.1 also has longer scaffolds than recent trio-hifiasm-based assemblies, but has shorter contig N50, lower QV, and substantially lower completeness."
7. Could the authors please elaborate on this conclusion "From a pilot study, an error-correction module of the 3D-DNA pipeline seemed to split long repetitive regions complicatedly, and it made difficult to construct scaffolds or curate misassembles (Fig. S1)"? No Fig S1 was included in the submission, and this

merits more discussion and detailed methods if the authors want to claim this.
8. The authors should state in the main text that the N50 read lengths from ONT and HiFi, since they are relatively small compared to current best practice.
9. It would be useful to compare to other recent reference genomes and assemblies, such as https://genomebiology.biomedcentral.com/articles/10.1186/s13059-020-02047-7, https://doi.org/10.1101/2021.06.10.447952, and https://www.nature.com/articles/s41592-020-01056-5

Level of Interest

Please indicate how interesting you found the manuscript: Choose an item.

Quality of Written English

Please indicate the quality of language in the manuscript: Choose an item.

Declaration of Competing Interests

Please complete a declaration of competing interests, considering the following questions:

- Have you in the past five years received reimbursements, fees, funding, or salary from an organisation that may in any way gain or lose financially from the publication of this manuscript, either now or in the future?
- Do you hold any stocks or shares in an organisation that may in any way gain or lose financially from the publication of this manuscript, either now or in the future?
- Do you hold or are you currently applying for any patents relating to the content of the manuscript?
- Have you received reimbursements, fees, funding, or salary from an organization that holds or has applied for patents relating to the content of the manuscript?
- Do you have any other financial competing interests?
- Do you have any non-financial competing interests in relation to this paper?

If you can answer no to all of the above, write 'I declare that I have no competing interests' below. If your reply is yes to any, please give details below.

I declare that I have no competing interests

I agree to the open peer review policy of the journal. I understand that my name will be included on my report to the authors and, if the manuscript is accepted for publication, my named report including any attachments I upload will be posted on the website along with the authors' responses. I agree for my report to be made available under an Open Access Creative Commons CC-BY license

(http://creativecommons.org/licenses/by/4.0/). I understand that any comments which I do not wish to be included in my named report can be included as confidential comments to the editors, which will not be published.

Choose an item.

To further support our reviewers, we have joined with Publons, where you can gain additional credit to further highlight your hard work (see: https://publons.com/journal/530/gigascience). On publication of this paper, your review will be automatically added to Publons, you can then choose whether or not to claim your Publons credit. I understand this statement.

Yes Choose an item.
