# Supplementary material for: KOREF_S1: phased, parental trio-binned Korean reference genome using long reads and Hi-C sequencing methods
Source: Gigascience. 2022 Mar 24;11:giac022. doi: 10.1093/gigascience/giac022 (PMC8952264; doi:10.1093/gigascience/giac022)
Supplement: giac022_Reviewer_2_Report_Original_Submission — Chaochun Wei -- 9/10/2021 Reviewed [file giac022_reviewer_2_report_original_submission.pdf]

**Reviewer Report**

**Title: KOREF_S1: the phased, parental Trio-binned Korean reference genome using long-reads and Hi-C sequencing methods**

**Version: Original Submission** **Date:** 9/10/2021

**Reviewer name: Chaochun Wei**

**Reviewer Comments to Author:**

This paper reported the construction of KOREF_S1v2, a reference genome for Korean or Eastern Asian, using long read sequencing platforms in addition to NGS sequencing and HiC sequencing platforms. A reference genome construction method was introduced to combine parent genomes to increase the quality of the final assembled genome. The constructed genome was assessed for its quality by comparing it to the existing KOREF genome and the human reference genome. The goal of this paper is to provide an accurate Korean reference genome. A few issues are listed below.
Major issues
1. Line 52, "GRCh38 … derives from a single individual, mostly based on Caucasian and African ancestry", the content was incorrect, and the sentence needs a revision.
2. Line 200, "the genes included 20,378 protein-coding genes with 166,570 transcripts, 46,973 lncRNAs and 17,535 pseudogenes.", the number of protein coding transcripts, 166,570, was much bigger than the number for protein-coding transcripts listed in GENCODE, which is about 87K. Please double check the numbers.
Minor issues
1. Line 234, "and it made difficult to construct" -->" and made it difficult to construct".

**Level of Interest**

Please indicate how interesting you found the manuscript: Choose an item.

**Quality of Written English**

Please indicate the quality of language in the manuscript: Choose an item.

**Declaration of Competing Interests**

Please complete a declaration of competing interests, considering the following questions:

- Have you in the past five years received reimbursements, fees, funding, or salary from an organisation that may in any way gain or lose financially from the publication of this manuscript, either now or in the future?

- Do you hold any stocks or shares in an organisation that may in any way gain or lose financially from the publication of this manuscript, either now or in the future?
- Do you hold or are you currently applying for any patents relating to the content of the manuscript?
- Have you received reimbursements, fees, funding, or salary from an organization that holds or has applied for patents relating to the content of the manuscript?
- Do you have any other financial competing interests?
- Do you have any non-financial competing interests in relation to this paper?

If you can answer no to all of the above, write 'I declare that I have no competing interests' below. If your reply is yes to any, please give details below.

I declare that I have no competing interests

I agree to the open peer review policy of the journal. I understand that my name will be included on my report to the authors and, if the manuscript is accepted for publication, my named report including any attachments I upload will be posted on the website along with the authors' responses. I agree for my report to be made available under an Open Access Creative Commons CC-BY license (http://creativecommons.org/licenses/by/4.0/). I understand that any comments which I do not wish to be included in my named report can be included as confidential comments to the editors, which will not be published.

Choose an item.

To further support our reviewers, we have joined with Publons, where you can gain additional credit to further highlight your hard work (see: https://publons.com/journal/530/gigascience). On publication of this paper, your review will be automatically added to Publons, you can then choose whether or not to claim your Publons credit. I understand this statement.

Yes Choose an item.
